# Assessing the Security of Campus Networks: The Case of Seven Universities

**DOI:** 10.3390/s21010306

**Published:** 2021-01-05

**Authors:** Rui Zheng, Hao Ma, Qiuyun Wang, Jianming Fu, Zhengwei Jiang

**Affiliations:** 1 Key Laboratory of Aerospace Information Security and Trusted Computing, Ministry of Education, School of Cyber Science and Engineering, Wuhan University, Wuhan 430072, China; zr_12f@whu.edu.cn (R.Z.); 15827331132@163.com (H.M.); 2 Institute of Information Engineering, Chinese Academy of Sciences, Beijing 100093, China; wangqiuyun@iie.ac.cn (Q.W.); jiangzhengwei@iie.ac.cn (Z.J.); 3 School of Cyber Security, University of Chinese Academy of Sciences, Beijing 100049, China

**Keywords:** campus network, network assets, threat intelligence, vulnerability detection

## Abstract

The network security situation of campus networks on CERNET (China Education and Research Network) has received great concern. However, most network managers have no complete picture of the network security because of its special management and the rapid growth of network assets. In this investigation, the security of campus networks belonging to seven universities in Wuhan was investigated. A tool called “WebHunt” was designed for campus networks, and with its help, the network security risks were found. Differently from existing tools for network probing, WebHunt can adopt the network scale and special rules of the campus network. According to the characteristics of campus websites, a series of functions were integrated into WebHunt, including reverse resolution of domain names, active network detection and fingerprint identification for software assets. Besides, WebHunt builds its vulnerability intelligence database with a knowledge graph structure and locates the vulnerabilities through matching knowledge graph information. Security assessments of seven universities presents WebHunt’s applicability for campus networks. Besides, it also shows that many security risks are concealed in campus networks, such as non-compliance IP addresses and domain names, system vulnerabilities and so on. The security reports containing risks have been sent to the relevant universities, and positive feedback was received.

## 1. Introduction

**Figure 1:**
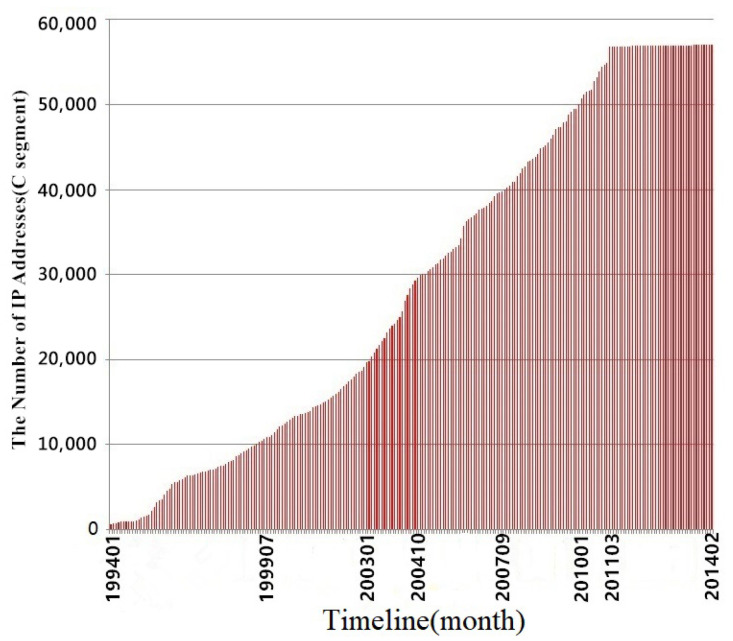
Statistics of IPv4 addresses assigned by CERNET (1994–2014) [5].

China Education and Research Network (CERNET) is a particular Internet network for education and research composed and constructed by universities [1]. As a subnet of CERNET, a campus network stores a lot of sensitive information and documents. Most services provided by universities are built into the campus network for students and the public. Besides the university’s official agencies, individuals and groups related to the universities also set up the website on the campus network. These factors enable increases in the scale and complexity of the campus network (as shown in Figure 1). With the addition of decentralized network management, the network managers find it hard to assess campus networks’ security. The campus networks often have security problems. In fact, campus networks are treated as a place for penetration exercises [2], which is also known from Google results (keywords are “university” and “hack.”).

Differently from the traditional Internet, the IP addresses and domain names of CERNET are proprietary for universities. The organizations and individuals related to a college or university apply to the campus network management department for subdomain names and IP addresses to build public Web services, which follows “The registration measure of CERNET EDU.CN network domain name” [3]. Non-standard use of the domain names and IP addresses belonging to CERNET perhaps trigger severe information security risks for the public or students, such as fraud utilized by faking an EDU.CN domain name, because the top-level domain name of “EDU.CN” has become an important symbol of colleges/universities in China [4].

The lack of security of campus networks has given rise to a serious information security emergency. Wannacry virus outbreaks on campus networks examples [6] that caused massive losses to the students and teachers of universities in China [7]. Therefore, it is urgent to discover these security risks in campus networks of colleges and universities. The detection of network assets and their potential threats has tremendous significance to colleges and universities’ network security.

For assessing the campus network’s security, the first thing is to make clear the network assets. Network asset detection refers to discovering and identifying network assets, which collect relevant information through network scanning. Based on the detection of network assets, compliance audits of domain names and IP addresses belonging to CERNET are performed. Simultaneously, the vulnerabilities contained in systems will be located through matching software version with vulnerability information. However, Existing studies [8,9,10] mostly combined vulnerability libraries with cyberspace device search engines to conduct non-invasive vulnerability evaluations, which have many weaknesses, such as detection methods not allowing real-time detection, and the cyberspace device search engine not being not customized. These factors will affect the efficiency and accuracy of the detection results. Like these theoretical methods, existing commercial tools [11] for network scanning are also not customizable. They cannot process the special resolving regulation and network structure of the campus network.

Based on solving the above problems, we investigated campus network security by taking seven universities as instances in Wu Han. A dedicated tool called WebHunt was designed and implemented in the investigation process by associating active network asset detection with threat intelligence. Unlike existing theoretical methods and commercial tools, WebHunt consists of several components designed for the campus network. Consequently, WebHunt can get more comprehensive security information of seven universities. Finally, reports of security information were sent to these universities’ network management departments, and positive feedback was received from them. Our main contributions are as follows:

(1) According to the campus network characteristics, a series of components are integrated into WebHunt, such as reverse resolution and compliance audits of CERNET domain names. The experiments show that the detection results of WebHunt for campus network are better than cyberspace device search engines’ results.

(2) By designing a set of synthetic fingerprints, software information is identified accurately. Manual analysis and comparative analysis prove the asset identification module’s effectiveness in WebHunt, which provides a good foundation for vulnerability identification.

(3) After probing the campus networks of seven universities in Wuhan of China, WebHunt found some security risks. These security risks have been reported to seven universities’ network security management departments, and positive feedback was received from them.

## 2. Related Work

As far as we know, this paper is the first study focusing on the security of campus networks belonging to CERNET. WebHunt is a combined tool that contains many components. In this section, several methods are described and compared around finding network assets and vulnerabilities. Firstly, we survey the main methods of network asset detection. Next, network service fingerprint technology and software vulnerability tools are described separately. Through comparing existing commercial tools and academic papers, the main functions of WebHunt we discussed are confirmed.

### 2.1. Network Asset Detection

The method of network asset information collection can be divided into a statistical method and a detection method. The statistical method depends mainly on statistic software [12,13], which is essentially a manual operation, so is time-consuming and has management costs. Obviously, The statistical method is not fit for the campus network’s size. The detection technology can obtain the network equipment and its connection information by deploying the server and analyzing traffic flow from the host. Therefore, this kind of technology is mainly used to obtain network asset information in the WebHunt.

The detection method can also be divided into passive detection and active detection according to whether it is necessary to construct data packets. Active detection identifies the port, system, service and application by establishing a connection with the target host and sending constructed special packets. Active detection can be implemented in various ways, such as a TCP three-way handshake [14] and ICMP ping [15]. These implementations can be divided into the full-link scan, semi-link scan and hidden scan depending on whether a complete three-way handshake link needs to be established. The specific classification is shown in Table 1.

Nmap [16] is a prevalent active detection tool for network asset detection, which achieves many functions, including host survival detection, port detection and service application identification by matching the characteristics of the response data packets corresponding to different customized data packets. However, Nmap is easy to be limited by the number of TCP connections of the TCP/IP protocol stack. Hence, the detection speed of Nmap is slow, and it is not suitable for large-scale network device scanning. DNmap [17] improved Nmap’s efficiency after introducing a distributed framework, but the target host still limits the number of TCP scan connections. Masscan [18] and Zmap [19] rebuild the scanning mechanism based on asynchronous stateless scanning, significantly improving the scanning rate. Masscan and Zmap apply to only port scanning and host discovery, without detecting operating systems, software services or applications. Active detection is an efficient method for asset detection. However, the notable drawback of active detection is that the firewall may filter it because of perhaps traffic jams.

Passive detection identifies specific services by collecting and analyzing traffic flow with probe devices deployed in specific network locations. The traffic flow must be enough for the identification of the specific service. The common passive asset detection tools are p0f [20], PRADS [21], Satori [22] and so on. The p0f [20] is a pure passive fingerprint identification tool, which achieves fingerprint recognition by sniffing and analyzing the data packets. The categories of information the p0f recognizes are poor. In order to cope with the surge of network traffic caused by the growth of network scale, Barnes et al. [23] proposed to deploy p0f in the Linux kernel space, which greatly speeds up analysis of network traffic. PRADS [21] passively listens to network traffic and gathers information on hosts and services. The information can map the network, letting the users know what services and hosts are active. Satori [22] identifies the network assets and their operating systems based on the DHCP message’s options and its order feature. The features of DHCP messages help Satori achieve high accuracy. However, the DHCP message is only visible on the LAN, which limits the application of Satori. Ding et al. [24] discovered common servers through analysis the traffic flow collected by servers deployed in CERNET, which only can identify four kinds of basic service, including Web, DNS, email and NTP. In a word, passive detection has less impact on the target host network, but the detection’s comprehensiveness and efficiency are limited by the richness of acquired traffic. Compared with the passive method, active detection can discover idle services in the target network by establishing a connection to idle services—the passive approach may miss these devices [25]. Most cyberspace device search engines use active detection as the main tool to acquire device information.

Common cyberspace device search engines include Shodan [26], Censys [27], ZoomEye [28], FOFA [29] and so on. These search engines, as services providing access to Internet users, consist of multiple probe servers deployed in the Internet that scan global cyberspace resources continuously, and store the collected data in a database. Shodan [26] is the world’s first and most popular cyberspace search engine, which focuses on asset discovery at the host level. It can retrieve type information about all devices and components connected to the Internet. ZoomEye [28] focuses on assets discovery at the Web level; it retrieves device fingerprints, Web services, etc., depending on the big data storage processing platform. Censys [27] scans the IPv4 addresses for querying the composition of the Internet with the aid of ZMAP. Each method of network asset detection has its advantages and disadvantages. Many related studies have described them in detail, which are shown in Table 2. We found that existing network asset detection tools need to have clear detection targets, such as IP address lists or domain name information. Nevertheless, it is difficult to acquire a complete target list of IP addresses and domain names for an intricate network in practice.

### 2.2. Fingerprint Extraction and Matching

Fingerprint matching is a method to determine the type of a host or a device. The targets of fingerprint identification include port service identification and Web application identification. Most of the existing Web server fingerprint tools focus on the feature value matching, such as “Server” in the header of the response packet [30]. Lee et al. [31] proposed a method of determining the type of HTTP server by requesting a URL sub-link that does not exist in the target system. The content of the Web page returned by the Apache server is “not found.” In contrast, the Web page’s content returned by Microsoft IIS is “not found object.” The difference could be evidence to identify Apache and Microsoft IIS.

Huang et al. [32] indicated that the detection method involved in this specific field has a natural weakness that the value of a specific field is easily falsified. The obtaining of more precise results depends on constructing the exception request. The acquisition of fingerprint has become key to identifying the server type. Yan et al. [33] used the code audit tool for the Web application to collect fingerprint information. Cao et al. [34] used the K-means to identify different network terminals with the feature of the number of fields in the response header. However, this method may get a high false-positive rate because a different Web server has the same number of fields in the response header. Nan et al. [35] proposed a Web server fingerprint scheme based on the KNN algorithm. Two different types of exception requests are constructed to identify the corresponding Web server type and version, but it does not support exact version number identification.

Besides the traditional machine learning models, deep learning models have been applied to network asset detection. Regarding network asset detection, Vera et al. [36] designed a website fingerprint method of deanonymizing for traffic based on deep learning. This method can automatically learn robust and flexible website fingerprint features through self-encoder and different deep neural network algorithms. In terms of content analysis, the RNN model could be also used in traffic analysis [37].

Most of the above research is for the detection of one type of Web application, without considering multiple applications, including Web server category, scripting language, CMS (Content Management System) and other third-party applications deployed on the server host. CERNET common server database designed by Ding et al. [24] only discovers four kinds of common servers. Moreover, specific field matching lacks resilience to adversary forgery. The methods based on machine learning need more computing resources, which is not friendly to massive traffic. The WebHunt avoids these disadvantages by designing a combined fingerprint method.

### 2.3. Network Vulnerability Assessment

The network vulnerability assessment is a kind of security service based on network asset intelligence. Network vulnerability assessments can be divided into intrusion detection and non-intrusion detection according to whether generating detecting traffic may disturb normal work.

OpenVAS [38] and Nessus [39] are popular vulnerability assessment tools that identify the device type and its exploitable vulnerability by sending specialized packets to the target. However, artificial traffic may cause potential damage to the target network, such as denial of service. For that reason, permission from the device owner is necessary before vulnerability assessment. Moreover, Nessus is not flexible. The specialized plug-ins for different OS are indispensable, and cost much because minor changes in the service description string (banner information) may cause tool failure.

The vulnerability detection tools based on cyberspace device search engines do not need to probe network assets; instead, they obtain network assets information by querying the existing database and analyzing the vulnerability data. Genge et al. [8] built a tool called shoVAT based on Shodan API. With the help of Shodan API, ShoVAT obtains a list of available services and service-specific information in the range of the target host IP list. These data can be used to build CPE (common platform enumeration) for identifying possible vulnerabilities. Montz et al. [9] compared many metrics between Censys and Shodan and found the results of Censys are more suitable for discovering the vulnerabilities. Simon et al. [10] combined the cyberspace device search engine with the search results of the Google Dork. This could achieve better vulnerability matching through pairing search results with a public vulnerability database. However, these studies rely on cyberspace search engines, which have a natural weakness that they are developed for assets that have public IP addresses. Obviously, this is not suited to local area network scenarios, such as some fraction of any campus network.

## 3. System Design

WebHunt consists of five modules, including network asset mining, network asset identification, vulnerability knowledge graph, discovery of security risk and visual monitor modules. The functions and technical details of these components will be described in this section.

**Figure 2:**
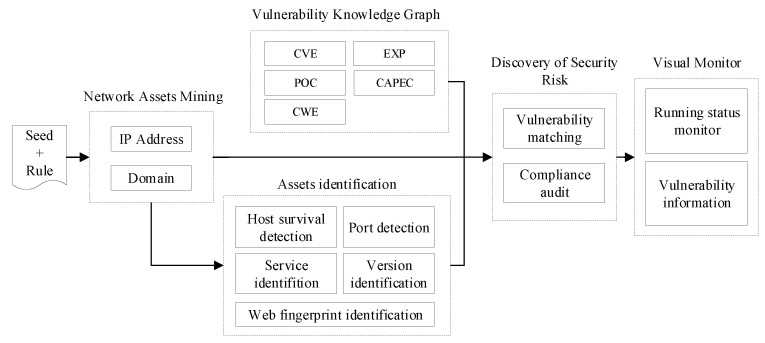
System framework diagram.

The five modules are shown in Figure 2. The network asset mining module mainly crawls the IP addresses and domain names for the network asset identification module according to predefined network seeds and detection rule. Based on the IP addresses and domain names, compliance audits are performed, and the network asset identification module outputs network assets details through host survival detection, port detection and other detection methods. The vulnerability knowledge graph module collects vulnerability information from the Internet and constructs a vulnerability database with the knowledge graph algorithm’s help. Assets information and vulnerability knowledge graph are associated with conducting vulnerability detection by the discovery of security risk module. The visual monitor module is displayed in the form of a Web interface, which provides windows to interact with users and interfaces for displaying asset statistics and vulnerability information.

Asset detection, risk analysis and monitoring network assets’ status are performed automatically after assigning specific URL seeds, rules and detection modes in WebHunt. The whole process does not affect the normal operation of the campus network service by setting scanning parameters, balancing between detection performance and running the target network system.

### 3.1. Network Asset Mining

A university applies for the first-level domain name from CERNET before establishing its campus network. The university’s network service is almost all bound to the second-level domain name under the first-level domain name for external access. The network assets in the campus network are messy. However, these devices generally have the domain characteristics of secondary institutions. In other words, some devices connect to each other through hyperlinks or the same IP address segments. According to these characteristics, WebHunt designed the network asset mining module.

**Figure 3:**
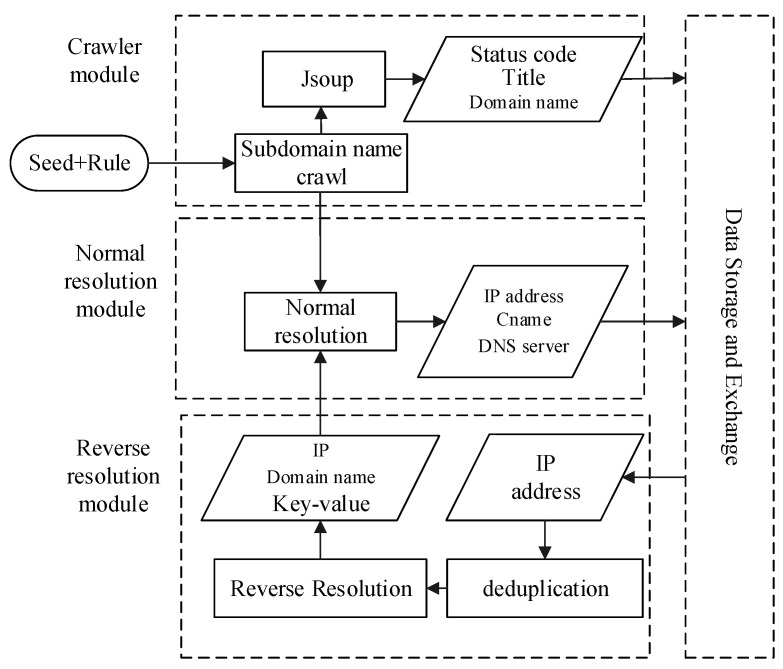
Flow diagram of network asset mining.

The network asset mining module provides a convenient and straightforward interface for users. After users enter seed links and rules according to the system settings, the multi-threaded crawler will start in the background to obtain the domain name asset. Figure 3 shows the flow diagram for network asset mining; keywords “Seed” and “Rule” denote crawl seed and crawl rules, respectively. A crawl seed is a starting point for crawling webpages. Considering the characteristics of the university node in CERNET, the seeds need to pick widely to ensure that the crawling is comprehensive enough. The keyword “Rule” indicates the rule of the crawler is obeyed. According to each university’s domain name particularity, the keyword “Rule” is generally indicated by the university’s top-level domain name. Take Wuhan University as an example; the rule is “whu.edu.cn” (Table 3).

The module of network asset mining traverses the five layers of the campus network’s link from a specified seed URL to the breadth-first traversal strategy. The network asset mining module’s crawlers will access each URL in the Web page and inspect its running status. For the eligible URLs, the crawlers record their domain names, status codes, website system titles and other information. Besides domain name crawling, domain name resolution is performed at the same time. Domain name resolution is a basic Internet service that converts a domain name to IP address commonly implemented by a public DNS server.

In the network asset mining module of WebHunt, as shown in Figure 3, a domain name’s resolution can be divided into forward resolution and reverse resolution. Forward resolution has been described as above. However, with the forward resolution it is easy to miss the domain names because of existing domain names bound to one IP address in the campus network. Reverse resolution is the opposite of forward resolution, which obtains one or more domain names through an IP address. More domain names bound to the same IP address are obtained in this process, which makes up for the defect that the webpage crawler only obtains limited domain names. Considering engineering approaches for building websites, such as load balancing and VPS (virtual private server), it is ubiquitous that multiple domain names are resolved to the same IP in practice. WebHunt performs forward resolution and reverse resolution alternately for network asset crawling comprehensively. The query of forward resolution is implemented with the aid of Microsoft’s command-line tool NsLookup program, while the query of reverse resolution is performed by the threat intelligence platform VirusTotal. VirusTotal obtaining reverse resolution records relies on the PDNS server maintained by Google. The PDNS server [40] has collected the information and stored it in a database, including domain names, IP addresses and their relationships, which together easily leads to a certain lag. For alleviating the accurate loss, WebHunt performs forward resolution again after reverse resolution and automatically preserves the results verified by forward resolution.

The module of network asset mining mainly obtains information, including domain name, IP address, access status, home page TITLE, resolution authority server, CNAME record, etc. According to tests for campus networks belonging to many universities, traversing the five layers of the network link and reverse analysis could cover most of the campus network’s domain names. Although other domain names can be obtained by traversing more layers, the number of rest domain names is extremely small, and the time consumption increases too much.

### 3.2. Network Asset Identification

The domain name obtained from the network asset mining module is far away enough for vulnerability detection. The status of the port, running service, service version, application component, etc., plays more critical roles in the security assessments of campus network assets. For instance, the software version number always indicates a vulnerability or one that has been repaired. As a consequence, more precise information about network assets is more important for vulnerability detection.

**Figure 4:**
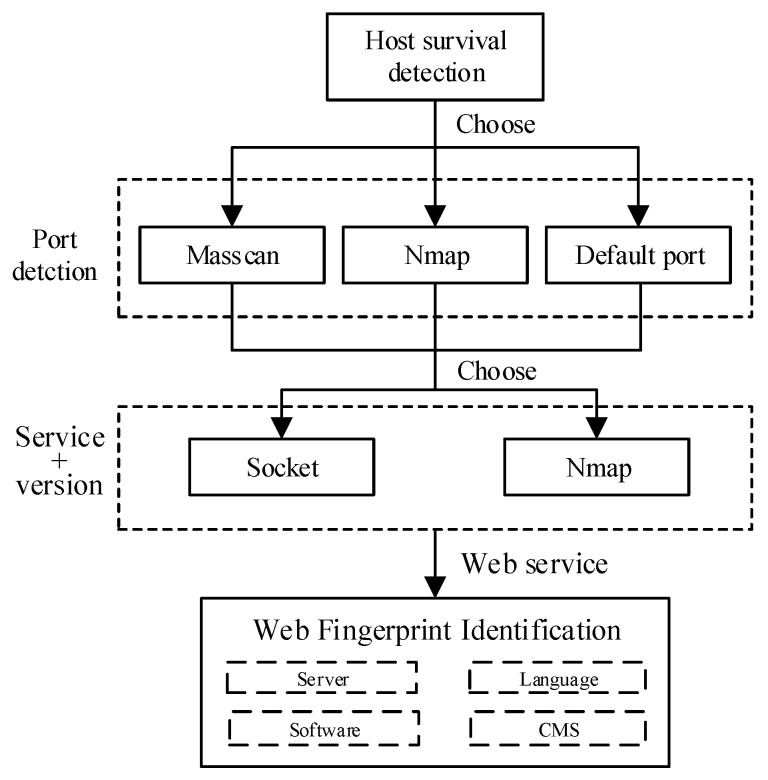
Network asset identification diagram.

As described in Section 2.1, network asset detection can be divided into passive detection and active detection. Passive detection for network assets depends on deploying probes in stem paths that are difficult for complicated campus networks. The performance of passive detection is far away from active detection considering comprehensiveness and efficiency. After considering the timeliness and accuracy of detection, WebHunt uses active detection to identify assets. The process is shown in Figure 4. Three functions of asset identification are described below.

**Host survival detection**. Host survival detection is an indispensable step before other network assets identify functions because port detection or services identified to invalid hosts are inefficient. Therefore, host survival detection can improve network asset detection efficiency by reducing the number of targets in the schedule. WebHunt implements host survivability detection by analyzing the differential echo responses of ICMP protocol data packets from the target host. ICMP messages constructed by WebHunt are sent to target hosts in the manner of multithread; then WebHunt verifies response messages and finally determines whether the detected host is alive.

**Port detection**. The port is a communication channel to interact with services or Web applications and an intrusion channel that can be easily exploited by hackers. Monitoring port status and closing unnecessary ports can reduce security risks. Moreover, the port information can provide a foundation for services and Web application fingerprinting. WebHunt provides various port detection methods for users according to the actual scale, detection inclination and other factors of the campus network. Masscan is preferentially used for port scanning in WebHunt. Masscan is a typical representative of the stateless port scanner, which uses the optimized target randomization algorithm for continuous IP segment scanning to avoid the IDS detection effectively in the target network. However, suitable bandwidth for port high-efficiency detection of Masscan is necessary, determining the setting of detection rate. Besides Masscan, Nmap is used as an extra port detector with high flexibility and powerful fingerprint libraries.

**Fingerprint recognition**. During service identifying, port detection results are not enough to make sure which service is running on it. Fingerprint matching is efficient for asset identification. Due to the vast applications of computer network technology, network services are becoming more and more diverse. It is necessary to extract an exclusive and special fingerprint feature for each type of network asset. Fingerprint identification includes discernment of port services and Web applications.

The identification of a port service can be implemented in three ways: banner information, constructing special packets and default ports.

(1) Banner information identification. The banner information may contain sensitive information, such as the software provider, software name or software version. Establishing a SOCKET connection can quickly identify these services and their versions according to the returned differential banner information.

(2) Special data packet construction. The specific data packets are sent to the specific port of the target host. The service is recognized according to the return information by listening to the host’s response mode and analyzing its content, which is fast and reliable.

(3) Default port. The default port setting is an important way to identify certain services. The default port setting will become significant evidence if the service cannot be identified by the previous two means or if the service does not change the port by default. For example, the default port number for DNS is 53, the default port number of NetBios (Network Basic Input/Output System) is 139 and so on.

The most effective feature for Web application identification is the fingerprint, which includes keywords in a webpage or a special file path. In order to improve the identification rate of Web applications, after inspecting open source and mainstream Web application code—CMS, Web server, third-party application software, etc.—five aspects of information of Web applications are used as fingerprint resources:

(1) Directory structure characteristics of Web applications. Differently Web applications have large gaps in structural design such that Web applications can be identified based on their specific directory structures. For example, the /wp-includes/ directory in the Web application’s file path can be used to determine that the application is WordPress. Some examples are shown in Table 4.

(2) Web application signature. Generally, Web applications have some signatures in HTML or JS, CSS files, etc., similarly to the technical principle of some intrusion prevention systems, such as WAF. Matching the keywords of the HTML source code is an important method for Web application identification. However, the disadvantage is the lack of robustness because the keywords are easy to modify or delete. Fortunately, we found that the keywords are rarely modified or deleted in campus applications after testing a campus network’s Web system. Hence, the Web application signature still is used as a supplementary rule for Web application identification in WebHunt. For example, the robots.txt contains the signature code such as “wp-admin,” and the webpage contains signature code such as “wp-content” path in some Web applications. These points indicate that WordPress is likely deployed on the host. Some examples of Web application signatures are shown in Table 5.

(3) Static files. For example, JS and CSS files are used for front-end layout design and dynamic interactions. JPG and PNG files are used for icons or page backgrounds. Web applications can be identified by these static files, which are used directly without modification. Some examples are shown in Table 6.

(4) Cookies. According to different cookies, Web applications can be accurately identified. For example, a set-cookie value including SQMSESSID can be used as evidence for the existence of SquirrelMail.

(5) Header information of the Web system. The header information of the response packet will indicate the particular Web application.

### 3.3. Vulnerability Knowledge Graph

The module of the vulnerability knowledge graph is responsible for processing vulnerability intelligence according to the characteristic of common vulnerability for the campus network. The vulnerability knowledge graph module will output the knowledge graph about vulnerability.

**Figure 5:**
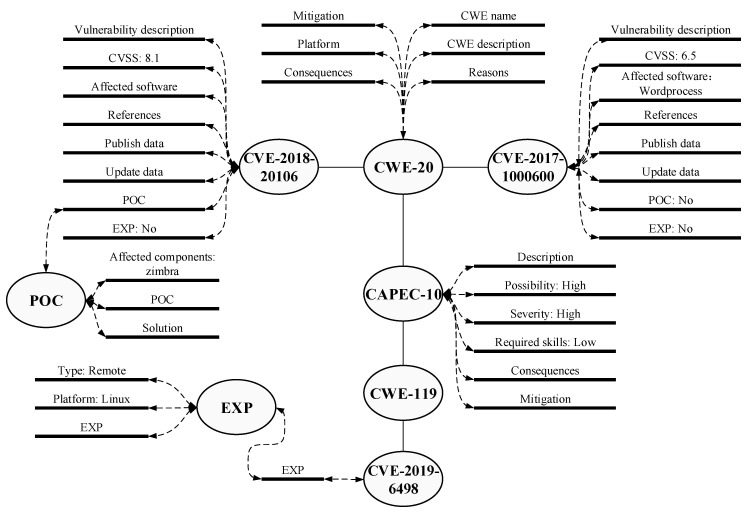
Vulnerability knowledge graph description.

Network assets’ vulnerability in campus networks can be divided into code defects in self-developed software programs and the vulnerabilities from third-party components. Vulnerabilities caused by code defects, such as SQL injection and cross-site scripting attacks in self-developed software systems, can be detected by the Web application vulnerability scanner. For the unrepaired vulnerabilities that have been made public on the Internet in the campus network, vulnerability scanning technology was developed built on the vulnerability knowledge graph in WebHunt. By matching network assets information and the local vulnerability database, the vulnerabilities are found, and WebHunt estimates further whether they can be attacked or exploited. WebHunt establishes and maintains a local vulnerability database that stores multi-dimension vulnerability information, including CVE, CWE, CAPEC, POC and EXP. These data sources are shown in Table 7, and one example of a vulnerability description is shown in Figure 5.

Vulnerability information collection depends on the self-develop crawler and public API organizer provided. Instead of a smooth public API, various problems, such as an anti-crawl protector, may arise in developing a self-develop crawler. Fortunately, most anti-crawl mechanisms can be bypassed by randomizing the request header and setting the proxy. However, these solutions are sometimes invalided. For example, Seebug deployes multiple anti-crawl strategies. We bypass Seebug’s client’s verification by obtaining veritable cookie information corresponding to “_jsluid” and “_jsl_clearance” generated by the selenium simulation browser.

WebHunt integrates almost all kinds of vulnerability information based on CVE standards in order to maintain the consistency and integrity of the vulnerability information database. Simultaneously, CWE, CAPEC, CVSS, POC and EXP are compatible with the CVE standard of vulnerability knowledge graph and supplement the vulnerability description. The vulnerability knowledge graph of WebHunt not only further indicates the degree of damage and exposure of vulnerability, but also establishes intrinsic links between different vulnerabilities. Besides, WebHunt provides two kinds of update strategy for asset identification: the full update and the incremental update. The full update is used to build a local vulnerability library when the WebHunt is first deployed. An incremental update obtains the new vulnerability information by creating the heartbeat threads, thereby monitoring the vulnerability data updates from sources. Every incremental update will trigger the detection once again for a possible new vulnerability in the campus network.

After the local vulnerability database update, the system automatically generates data and immediately draws on the vulnerability knowledge graph. The graph is drawn with the aid of D3 visualization technology [41], a professional visualization tool that can be used to achieve a knowledge graph. These entities in the local vulnerability database are taken as different points, and the relationships between entities are taken as lines to draw a force-directed graph. Therefore, the force-directed graph will associate different CVE values belonging to the same CWE and the different CWE values belonging to the same CPE. Besides, the POC and EXP will correlate with the specific CVE. In addition, WebHunt provides a retrieval interface for the details of the vulnerability analysis in the database.

### 3.4. Discovery of Security Risk

The discovery of security risk module is responsible for locating security risk, which has two main functions of finding vulnerability information of network assets and auditing compliance of domain name and IP address.

**Associating network assets with vulnerability**. The discovery of security risk module detects software system vulnerability by matching the campus network assets and local vulnerability database. The association analysis uses a combination of exact matching and fuzzy matching. Exact matching can identify the vulnerability of the software system in the campus network through matching software version. For instance, WebHunt can detect vulnerability of CVE-2017-7269 when Microsoft IIS 6.0 is deployed on the server because the knowledge graph recorded that IIS 6.0 has a strong relation to CVE-2017-7269. Instead of the software version, fuzzy matching focuses on the software name, which retrieves all the relevant software vulnerabilities information and supplements the exact matching. In other words, fuzzy matching provides information for understanding software vulnerabilities from a macro perspective, such as SMB always exposes high risk vulnerability in Windows Server.

**Compliance audit**. The irregular use of IP addresses and domain names in the campus network may lead to security risks. The problems that the compliance audit solved mainly include the following aspects:

i. IP address and domain names of CERNET and public network mixed. Domain name belonging to CERNRT is bound to the public network’s IP, or the public domain name is bound to the CERNET IP, which does not comply with the CERNIC regulations.

ii. Domain names bound to campus intranet IP are still resolvable outside the CERNET, which results in the leakage of Intranet addresses.

iii. Domain name access error will affect the service for students and the public. In particular, abandoned CERNET domain names can still be resolved on the DNS, which may be abused for malicious purposes.

iv. Improper management of CNAME may cause the hijack vulnerability of the subdomain name.

v. Domain name resolution error results in resource wasting and other issues.

### 3.5. Visual Monitor

The visual monitor module mainly presents the system information through the webpage, including the statistical information of campus network assets, the running status of each campus network asset, the alarms for asset vulnerability information and the mapping of the vulnerability knowledge graph. In addition to this, the visual monitor module also provides an interactive interface for users.

In order to monitor continuous changes of network assets, WebHunt sets heartbeat threads, deletion threads. Heartbeat threads are used to perceive network asset changes during detection tasks and synchronize the latest asset information. Deletion threads are used to monitor the change of service current status. In the visualization module, WebHunt shows the statistics of asset changes, including new, updated and closed services in the campus network every day. WebHunt sets up a special module to record the historical asset information, which helps network managers track back the original asset status and record the change process of services.

## 4. Evaluation and Investigation

Network security investigation of seven universities will be described in this section. Evaluation of the WebHunt is also performed by comparing with other network tools. In the first subsection, the experimental scheme is described; In the second section, the performance of capturing assets by WebHunt and other tools is presented, this is the basis of the investigations. In the following sections, WebHunt is used for compliance analysis, the exact identification of network assets and services, vulnerability discovery, and so on. At last, a summary of the campus network’s security about these universities is performed.

### 4.1. Background

In this section, network security’s investigation is performed on campus networks of seven universities, including Wuhan University (WHU), Huazhong University of Science and Technology (HUST), China University of Geosciences (CUG), Central China Normal University(CCNU), Huazhong Agricultural University (HZAU), Zhongnan University of Economics and Law (ZUEL) and Wuhan University of Technology (WHUT). These universities all have a top-level domain name in CERNET and own a massive amount of network assets. Among these top-level campus network systems, we focused on analyzing the three largest universities’ network security situation with the aid of WebHunt. These three largest universities are Wuhan University (WHU), Huazhong University of Science and Technology (HUST), and China University of Geosciences (CUG).

We evaluate the performance of WebHunt from three aspects of network asset acquisition: compliance auditing, network asset identification and vulnerability match results, which are the main functions of WebHunt. First, we analyze the asset list’s acquisition to verify our method’s effectiveness for the situation of many domain names bounding to the IP address. After that, the IP address and domain name’s compliance audit results are listed to determine whether there are irregular uses and potential security risks. Finally, we compare the results of asset identification by WebHunt with the cyberspace search engine and show the correlation between network assets and vulnerabilities.

### 4.2. Network Asset Acquisition

The acquisition of network assets is the foundation of WebHunt, which directly affects the following functions’ performances. An example of seeds and rule WebHunt used are shown in Table 3. The seed links need to be scattered as far as possible to obtain more extensive network assets information. In the experiment, we selected the homepages of universities’ secondary institutions as seed links, which can be obtained from the institutional settings page of universities’ homepages.

After mining the network assets of three universities, the summary of network assets was listed in Table 8. The numbers of IP address and domain name have been de-duplicated. Domain names contain top-level domain names and lower level subdomain names. As seen from the table, each university has far more domain names than IP addresses. The reason is as before: many network services reused IP address resources. More subdomain names mean more comprehensive network asset mining. The results of domain name detection by four tools are shown in Table 9.

In order to verify the capability of WebHunt’s module for network asset mining, popular cyberspace search engines including Shodan, FOFA and ZoomEye have participated in the experimental evaluation as a control group. In the experiment, each university’s domain name was retrieved according to the specific rules of these cyberspace exploration engines, and the retrieval results are shown in Table 9 below.

In Table 9, the results of FOFA are obtained by placing the retrieval mode on “all.” The results contain all the records of the previous five years, which may include reduplicative data. When the year was set as 2019, FOFA obtained results of 328, 229 and 156 for three universities, respectively. Obviously, WebHunt has better results and more comprehensive detection in network asset detection compared with other public cyberspace detection tools.

The principle of cyberspace device search engine is that utilizing the probe server deployed in global to scan the network assets connecting to the Internet, and then the results are stored in the database for users query. In other words, the detection provided by cyberspace device search engine cannot be customized. The results of the search have essentially existed data in the database. Besides, this service mode has a certain lag and has no way to reflect network assets’ current information. In contrast to these public services, WebHunt can perform device detection whenever the users need it.

It should be noted that there are many IP addresses for Intranet services in the campus network, such as the specific function server that does not open port to the public network, which is difficult for cyberspace device search engines to crawl. While WebHunt provides a configuration interface for this type of internal inspection.

### 4.3. Compliance Audit

Differently from the Internet, campus networks manage independently but open to the Internet. As a result, campus networks apply to more compliance regulations. WebHunt can audit the use of CERNET IP address and domain name. In the experiment, we analyzed a variety of campus network situations with abnormal use, such as unresolvable errors, non-compliant relationship between domain names and IP addresses belonging to CERNET or Internet. In the end, we analyzed the causes of compliance problems and their security risks.

Domain names and IP addresses in the three universities campus network detected by WebHunt are listed in Table 10. The CERNET domain names of Wuhan University are resolved as LAN address for 40, the Internet domain names are resolved as LAN IP for 7. The domain names connected to the public network are resolved on the LAN IP address, which can be interpreted as leakage Intranet IP address. Simultaneously, 5 CERNET domain names are resolved to the Internet IP, ten public domain names binding CERNET IP address. According to the regulations of CERNIC, both of the two cases are non-compliant. The domain name of Huazhong University of Science Technology cannot be resolved for a total of 23. The other two universities also exist in this situation of IP parse error.

**Figure 6:**
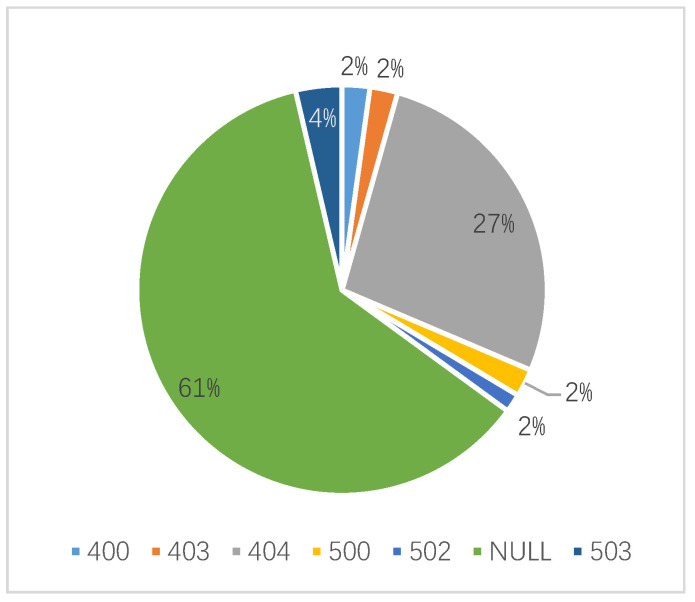
Distribution of status code of access error for domain names of Wuhan University.

For Wuhan University, the domain name’s resolution rate is 98%, which the resolution rate of domain name belongs to CERNET is 99.7%, and the resolution rate of the public network was 75.6%. The proportion of domain names belongs to CERNET and public networks that normally access is 76.2% and 48.8%, respectively. The difference between the two sets of data above indicates that some domain names access error. WebHunt obtains the corresponding IP addresses and classifies them according to the HTTP status code returned. As shown in Figure 6, the error codes of 400, 403, 404, 500, 502, 503 and NULL are found for Wuhan University. As known from the error code distribution, the access errors contain both client side and server side. not only client side but also server side error and cannot process requests normally. Through analysis of detection results, HUST and CUG have the same error exist.

The reasons for the access failure can be divided into three situations. The first one is these domain names no longer provide services. For this case, the network manager should submit a cancellation application for the domain names, and cancel the domain names resolution service, delete the Web data saved on the server. The second one is that the domain names can provide service, but downtime or other conditions result in abnormal access. The operations engineer needs to report in time and repair the error. Third, the access control strategy is assigned to restrict the access of some users. Even if WebHunt does its best to bypass these access control policies, it is impossible to avoid this situation entirely. After manual verification and analysis, the first and second abnormalities are relatively common. With the help of WebHunt, network managers grasp timely the status of assets in the campus network, which is unreachable for the cyberspace search engines.

CERNET domain name and IP address have a series of use restrictions, but the cross resolution of two types of network resources is still not completely avoided in practice. The CNAME record maps CERNET domain name to Internet domain name, which may cause the risk that CERNET users are accessing the malicious server. In this case, the public domain name is expired, but its CNAME record is not deleted, which will be exploited by criminals to execute subdomain name hijacking attacks through squatting the Internet domain name.

### 4.4. Network Asset Identification

The functions of port detection and fingerprint identification are built into WebHunt to ascertain the type of service, which provides software asset information for the association of assets and vulnerability modules. The network assets identification performed by WebHunt includes network service and Web application identification. Network services are identified by inspecting IP address resource, and Web applications are identified by inspecting IP address resource and domain name resource.

The results of network service detection by WebHunt (W), ZoomEye (Z), FOFA (F), or Shodan (S) are shown in Table 11. The symbol “-” means no detection or retrieval, which means same in subsequent tables. Obviously, the results of service detection from WebHunt is more comprehensive. More than 20 kinds of network services are detected; even the variants of protocols based on SSL protocol are identified, such as SMTP and SMTPS. The service types retrieved by ZoomEye include HTTP, Imap, POP, and FTP, but the quantity of hosts belongs to each type is less than WebHunt; FOFA is unable to provide direct retrieval of port service types. The request consists of one IP address or a segment of C IP address need to be submitted several times through the API or retrieval window. The results are not complete reports, which mean more cost to process results; Shodan retrieved more service types through host: “whu.edu.cn” than other cyberspace search engines, but not include basic service types such as Telnet, SMB and mysql. The overall comparison shows that WebHunt has a better performance in port service detection. Although some services have not been detected, such as NTP, Portmap and DNP3, it is because WebHunt does not have the identification rules for this service. In the future, more identification rules for services can be added to achieve more comprehensive service detection.

As all the experiments were performed in a real network environment, and the ground truth is hard to be labeled, manual verification was necessary for the results of WebHunt. Some network services deployed on remote hosts are difficult to identify by manual verification, so we selected partial services as the target of manual verification. The results are shown in Table 12. The number in brackets denotes the service number found by WebHunt. The number out the brackets denotes the service number of manual verification from the services found by WebHunt. Taking the result of manual verification as ground truth, the accuracy of WebHunt almost reached 100%. There were two false positive samples for DNS service, where the hosts open port 53 but do not provide domain name resolution service. The default port of the domain name server is not changed commonly. WebHunt adopts the default port identification method for DNS service. Besides, WebHunt found that a host belongs to a certain university provided VNC service allows log in anonymously, which has a huge security risk.

Web servers and Web applications are identified through fingerprint comparison. The results of the Web server and Web application recognition are shown in Table 13 and Table 14 respectively. As shown in Table 13, Web servers identified by way of domain name are greater in number than those found by other methods or cyberspace device search engines. The results show that WebHunt, which utilizes the IP address and domain name simultaneously to determine the type of software system, can effectively make up for the defect that insufficient coverage by way of IP resource only. Obviously, according to Table 13 the quantity of Web servers Shodan detected is the least; not only the categories of Web servers but also the number of Web servers detected are low. The results of FOFA are good for common types of Web server platform, but the coverage range of Web server types is shallow. The results of Zoomeye are close to the results from the domain name of WebHunt in kinds of the Web server and their quantity. We select Zoomeye and WebHunt’s domain name detection for analysis.

WebHunt can identify Web servers that rare used compared to Zoomeye, such as VappServer, Axis and Resin. However, there are two types of Web servers that WebHunt cannot recognize—Squid and Kangle; Zoomeye is reachable. The number of Tomcat servers identified by WebHunt system is far less than the numbers of ZoomEye and FOFA detections. After artificial analysis, Tomcat, as a Java application server, can be regarded as an extension of Apache and running independently for Apache. WebHunt did not distinguish the Tomcat server from the Apache server, and some Tomcat servers were identified as Apache servers. The literature [24] can only identify four kinds of CERNET basic server, far fewer than WebHunt.

Table 14 lists the Web applications such as content management system (CMS) and the other third-party applications identified by WebHunt and Zoomeye. Compared with Zoomeye, WebHunt identified most CMS types. However, for editors such as FCKeditor, identification of WebHunt is not effective. So far, the WebHunt system can recognize 81 port services, 22 CMS categories, 15 kinds of Web server, 4 scripting languages and 16 other Web application services.

### 4.5. Asset and Vulnerability Correlation Results

Based on the information of network assets, the local vulnerability knowledge graph is used to locate the vulnerability in the software system. The local vulnerability database data are updated several times every day, and the knowledge graph of vulnerabilities is built automatically. During the test, the data stored in the vulnerability database was limited to the disclosed vulnerability data after 2012. The data volume of the vulnerability database as of 9 April 2019, is shown in Table 15.

The vulnerability information in the software system will be presented in the Web interface, which performs exact matching and fuzzy matching information, specific asset information, details of the vulnerability and patch suggestions. The information of partial vulnerable assets in campus network matched accurately by WebHunt are shown in Table 16. It indicates that there are vulnerabilities in the Web server, including IIS6.0, IIS7.0, Nginx1.10.0, etc. The detailed information of these assets will be submitted to the network department of the university instead of disclosing here. Some software systems are identified for the types and applications without specific version information in the detection process. For this case, the fuzzy matching of vulnerability was applied to record the information and prompt associated risk messages to users.

The distribution of asset-related vulnerabilities is shown in Table 17. WordPress and PhpMyAdmin are two kinds of popular Web applications for campus networks, but there are more vulnerabilities than any application. Therefore, more attention should be paid to avoid this type of security risk. Zoomeye provides correlation between asset and vulnerability information without details, similar to the fuzzy matching of WebHunt. Hence, its result for vulnerability correlations is meaningless.

### 4.6. Summary

From the charts and figures in the above subsections, the campus networks’ security situations are terrible. Many vulnerabilities exist in campus networks, and non-compliance domain names and IP addresses can be utilized by hackers at any time. These security risks are the threats to information and documents in campus networks. As shown in the Table 18, these problems exist in almost all the universities investigated. Therefore, it can be inferred that the security condition of CERNET needs more attention from relevant departments. From Table 18, the problems at Wuhan University are relatively severe. On the one hand, Wuhan University has a long history, and the network device management involves many problems carried over from the past; on the other hand, it also reflects the loopholes in network management.

## 5. Conlusions

In this paper, we developed WebHunt according to the campus network topology characteristics, which integrate network asset detection, network compliance audits, vulnerability matching and other functions. With the aid of WebHunt, we found many security problems. These problems were distributed in seven universities, and some vulnerabilities even led to anonymous access. These results show that WebHunt has better performance than other Web scanning tools. These network security problems were reported separately to the universities, and we received positive responses.

## Figures and Tables

**Table 1:** Comparison of scanning technology.

Scanning Technology	Work Flow	Advantages
Full-link scan	A complete connection of three-way handshakes is established	High accuracy and stable scanning results
Semi-link scan	First two handshakes of TCP 3-way handshake are completed and the third handshake is interrupted	High scanning efficiency and low time consumption
Hidden scan	Other protocols which does not contain any part of the TCP/IP three-way handshake connection at all	Hidden scan is concealed and can bypass intrusion detection system, firewall, etc.

**Table 2:** Comparison of network asset detection methods.

Methods	Scope	Characteristics	Disadvantage
Manual statistics	Intranet, small scale	Information of network assets is comprehensive	Time-consuming and laborious
Client statistics	Intranet, small scale	The client collects and reports asset data automatically	Strong invasiveness and many limiting factors
Active detection	Fully applicable	Fast, fit for network assets that don’t generate traffic	High noise, easy to trigger an alarm
Passive detection	Intranet	Small invasiveness, supporting the accumulation of historical data	Invalid for assets that do not generate network traffic
Search engine	Public network	Search queries, fast, supporting the accumulation of historical data	Not fit for intranet, easy to be deceived

**Table 3:** Parameters of a network asset detection example for Wuhan University.

Seed	Rule
www.whu.edu.cn/jgsz/zjqtzz.htm www.whu.edu.cn/jgsz/znbm.htm www.whu.edu.cn/jgsz/zsdw.htm www.whu.edu.cn/jgsz/yxsz.htm www.whu.edu.cn/yhtd2/xs.htm www.whu.edu.cn/yhtd2/jg.htm www.whu.edu.cn/yhtd2/xy.htm www.whu.edu.cn/yhtd2/ks.htm www.whu.edu.cn/yhtd2/fk.htm www.whu.edu.cn	whu.edu.cn

**Table 4:** Directory characteristics of Web applications.

Structure Characteristics	Web Application
/wp-includes/	WordPress
/mspace/default1/	Discuz
/themes/bartik/color/	Drupal
/e/admin/	EmpireCMS
/phpmyadmin	PhpMyAdmin

**Table 5:** Signatures of text belonging to Web applications.

Files	Keywords	Web Application
/robots.txt	Emlog	Emlog
/fckeditor/fckconfig.js	Aspcms	AspCMS
/	discus!	Discuz!
/index	Zimbra	zimbra

**Table 6:** Static file characteristics of Web applications.

Files	Web Application
/joomla.xml	Joomla
/images/post/DhtmlEdit.js	Dvbbs
/wp-admin/images/wp-logo-2x.png	WordPress
phpmyadmin/favicon.ico	PhpMyAdmin

**Table 7:** Sources of vulnerability information collection.

Data	Source Address	Organizer	Collection Method
CVE	https://nvd.nist.gov/vuln/data-feeds	NVD	API
EXP	https://www.exploit-db.com/	EXPLOIT	Crawler
POC	https://www.seebug.org/	Seebug	Crawler
CAPEC	http://capec.mitre.org/	MITRE	API

**Table 8:** Numbers of domain names and IP addresses acquired for three universities by WebHunt.

Subject	Number of Domain Name	Number of IP Address
WHU	580	124
HUST	362	84
CUG	189	43

**Table 9:** Numbers of domain name assets acquired for three universities by WebHunt, Shodan, FOFA and Zoomeye.

Subject	WebHunt	Shodan	FOFA	Zoomeye
WHU	580	71	686	252
HUST	362	65	396	140
CUG	189	-	231	88

**Table 10:** Compliance audit of IP addresses and domain names.

Subject	Domain Names	Total	Access Normal	The Number of IP Address Resolved			Fail Resolved
				Intranet IP	Internet IP	CERNET IP	
WHU	CERNET	580	442	40	5	529	6
	Public	41	20	7	15	10	9
HUST	CERNET	362	285	0	1	341	20
	Public	46	36	0	19	24	3
CUG	CERNET	189	157	0	2	182	5
	Public	213	117	0	202	8	2

**Table 11:** Results of port service detection by WebHunt, Zoomeye, FOFA and Shodan.

Services	Results (W,Z,F,S)	Services	Results (W,Z,F,S)
Web	(263,57,-,30)	NetBios	(18,-,-,-)
VNC	(2,-,-,-)	Mysql-secured	(2,-,-,-)
Telnet	(5,-,-,-)	Mysql	(7,-,-,-)
SVN	(3,-,-,-)	ldap	(2,-,-,-)
SSH	(47,-,-,3)	Imaps	(6,-,-,4)
SMTPS	(4,-,-,4)	Imap	(5,6,-,6)
SMTP	(7,-,-,5)	FTP	(24,2,-,2)
SMB	(21,-,-,-)	DNS	(8,-,-,1)
Rsync	(1,-,-,-)	Docker-daemon	(1,-,-,-)
PPTP	(3,-,-,-)	DNP3	(-,-,-,1)
POP3	(6,6,-,5)	NTP	(-,-,-,1)
PortMap	(-,-,-,1)		

**Table 12:** Manual verification for network asset identification by WebHunt.

Services	VNC	Telnet	SVN	SSH	SMTPS	SMTP	POP3	Mysql	FTP	DNS
**Number**	2(2)	5(5)	3(3)	47(47)	4(4)	7(7)	6(6)	7(7)	24(24)	6(8)

**Table 13:** Numbers of Web servers detected by WebHunt, Shodan, FOFA and Zoomeye.

Web Server	WebHunt		Zoomeye	FOFA	Shodan
	IP Identification	Domain Name Identification			
Apache	105	237	80	174	11
IIS	41	57	66	23	3
WWW Server	6	38	19	-	-
VWeb Server	2	100	40	77	-
Tomcat	1	1	13	38	3
Resin	2	1	-	-	-
Nginx	30	47	19	-	6
VappServer	-	6	-	-	-
Axis	-	9	-	-	-
Squid	-	-	2	-	-
Kangle	-	-	2	-	-

**Table 14:** Numbers of Web applications detected by WebHunt, Shodan, FOFA and Zoomeye.

Web Application	WebHunt	Zoomeye
WordPress	50	4
PhpCMS	5	1
joomla	77	-
EmpireCMS	27	-
Discuz	1	1
zimbra	2	-
PhpMyAdmin	53	1
DedeCMS	12	5
AspCMS	1	-
Powereasy	2	-
FCKeditor	-	15
Drupal	-	1

**Table 15:** Data volume of local vulnerability library.

Vulnerability Database	Data Volume
CVE	63,805
POC	789
EXP	3811
CWE	715
CAPEC	518

**Table 16:** Number of vulnerabilities belonging to the Web servers and the number of these Web servers.

Web Server	Number of Vulnerabilities	Number of Assets
IIS6.0	1	18
IIS7.0	1	1
Ngix1.10.0	5	2
Ngix1.10.3	3	2
Ngix1.14.0	3	11
Php7.1.1	51	2
Resin3.0.28	1	1
Resin3.0.19	1	2

**Table 17:** The vulnerabilities detected by WebHunt.

Services	Number of Vulnerabilities	Services	Number of Vulnerabilities
IIS	4	Rsync	7
EmpireCMS	7	SMB	2
Ngix	17	SMTP	1
IMAP	4	SSH	1
IDAP	2	Tomcat	128
PHP	250	WordPress	131
PhpCMS	3	ASPCMS	2
PhpMyAdmin	136	DedeCMS	25
Resin	1	Axis	2

**Table 18:** The network security risks of seven universities found by WebHunt.

Security Risk	WHU	HUST	CCNU	CUG	HZAU	ZUEL	WHUT
Zombie url	√	√	√	√	√	√	√
Domain name access error	√	√	√	√	√	√	√
Cname record	√	√	√	√	√	√	√
LAN IP leakage	√	-	√	-	-	√	-
Domain name resolution disorder	√	√	√	√	√	√	√
Subdomain name non-compliance	√	-	-	-	-	-	-
Port open abnormal	√	-	√	-	-	-	√
CVE vulnerability	√	√	√	√	√	√	√
